# *Didymocarpus xuanlienensis*, a new species of *Didymocarpus* (Gesneriaceae) from Vietnam

**DOI:** 10.3897/phytokeys.275.185766

**Published:** 2026-05-21

**Authors:** Van Thi Thuy Tran, Son Thanh Hoang, Wen-Guang Wang, Xiao-An Lang, Hou-Cheng Xi

**Affiliations:** 1 Institute of Earth Sciences, Vietnam Academy of Science and Technology, Ha Noi, Vietnam Silviculture Research Institute, Vietnamese Academy of Forest Sciences Ha Noi Vietnam; 2 Silviculture Research Institute, Vietnamese Academy of Forest Sciences, Ha Noi, Vietnam Xishuangbanna Tropical Botanical Garden, Chinese Academy of Sciences Mengla China; 3 Xishuangbanna Tropical Botanical Garden, Chinese Academy of Sciences, Mengla, Yunnan, China Institute of Earth Sciences, Vietnam Academy of Science and Technology Ha Noi Vietnam; 4 Nanning Botanical Garden, Nanning, Guangxi, China Nanning Botanical Garden Nanning China; 5 Nanning Garden Expo Park Management Center (Nanning Institute of Tropical Botany), Nanning, Guangxi, China Nanning Garden Expo Park Management Center (Nanning Institute of Tropical Botany) Nanning China

**Keywords:** *
Didymocarpus
*, new taxon, taxonomy, Vietnam

## Abstract

*Didymocarpus xuanlienensis*, a new species from Vietnam, is described and illustrated here. It is morphologically similar to *D. brevipedunculatus* and *D. purpureobracteatus* in terms of corolla size and color but differs from *D. brevipedunculatus* in leaf blade shape, bract shape, indumentum, and calyx shape and differs from *D. purpureobracteatus* in peduncle indumentum, bract shape, and calyx shape. A detailed morphological description of the new species and a key to all *Didymocarpus* species in Vietnam are provided.

## Introduction

The genus *Didymocarpus* Wallich (1819: 378) (Gesneriaceae) currently comprises more than 100 species, distributed from India through the Himalayas to southern China, Indochina, and Sumatra (Rhuthuparna and Gowda 2024; Yang et al. 2024; POWO 2026). Historically, however, *Didymocarpus* included approximately 200 species before Weber and Burtt (1998) redefined the genus and segregated its species into three morphologically distinct genera: *Didymocarpus* s.s., *Henckelia* Sprengel, (1817: 402), and the newly established *Hovanella* Weber & Burtt, (1998: 333). More recently, Liu et al. (2025) reconstructed the molecular phylogeny of *Didymocarpus* and erected a new genus, *Palmatiboea*, to accommodate Southeast Chinese species formerly placed in *Didymocarpus*. In Vietnam, seven species and one variety of *Didymocarpus* are currently recognized (Tran et al. 2020; Middleton 2023; POWO 2026).

During field surveys in Vietnam on 2 December 2024 and 22 December 2025, an unidentified *Didymocarpus* species was collected in Xuan Lien National Park, Thuong Xuan Commune, Thanh Hoa Province. Based on comparative morphology, literature review, and examination of relevant herbarium specimens, it was confirmed that it represents a new species, which is described and illustrated below. A key to all known *Didymocarpus* species in Vietnam is also provided.

## Materials and methods

Plant material of the putative new species was collected from Xuan Lien National Park, located in Thuong Xuan Commune, Thanh Hoa Province, Vietnam. Type specimens have been deposited in the herbarium of the Vietnamese Academy of Forest Sciences (VAFS). Taxonomic descriptions are based on measurements taken from both fresh and dried specimens. Field surveys were carried out in the type locality and surrounding areas, and the conservation status was assessed following the IUCN Red List Categories and Criteria (IUCN Standards and Petitions Committee 2024).

## Taxonomic treatment

### Didymocarpus xuanlienensis

Taxon classificationPlantaeLamialesGesneriaceae

T.S.Hoang, W.G.Wang & H.C.Xi sp. nov.

96DC8AFF-7008-5FBE-9E6E-7AD45227D514

urn:lsid:ipni.org:names:77380512-1

Fig. 1

#### Type.

Vietnam • Thanh Hoa Province, Thuong Xuan Commune, Xuan Lien Nature Reserve, in evergreen forest on wet rocks, 574 m a.s.l., 20°01'N, 105°02'E, 22 December 2025, *Son Thanh Hoang, XL5316* (holotype: VAFS; isotype: VAFS).

#### Diagnosis.

The new species differs from the most similar species, *Didymocarpus brevipedunculatus* Y.H.Tan & Bin Yang, (2019: 191), in leaf blade elliptic to oblong, base cuneate (vs. ovate, base extremely obliquely cordate); bracts broadly ovate, glabrous (vs. orbicular to ovate, sparsely villous); calyx broadly campanulate, lobes triangular (vs. campanulate, lobes ovate to semiorbicular). *D. xuanlienensis* is also similar to *D. purpureobracteatus* W.W.Smith, (1912: 153) but differs from it in peduncle glandular-pubescent (vs. glabrous); bracts free (vs. often connate at base); calyx actinomorphic, broadly campanulate, lobes triangular (vs. slightly zygomorphic, tubular campanulate, lobes semiorbicular). Moreover, the new species differs from *D. brevipedunculatus* and *D. purpureobracteatus* in distribution, elevation, and flowering time. A detailed morphological and ecological comparison of these three species is presented in Table 1.

**Figure 1.**
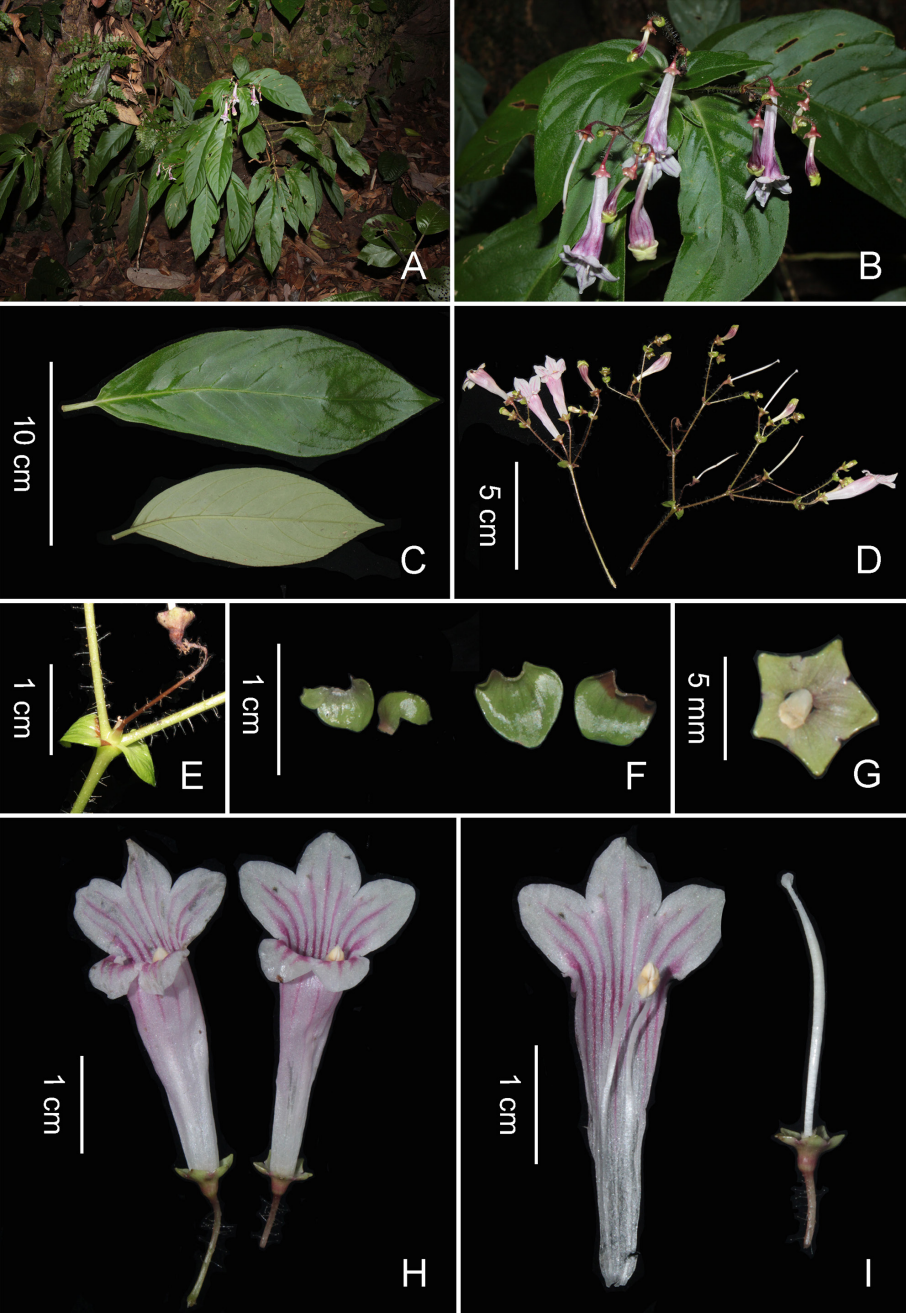
*Didymocarpus xuanlienensis* T.S.Hoang, W.G.Wang & H.C.Xi, sp. nov. **A**. Habitat; **B**. Flowering plant; **C**. Leaves, adaxial and abaxial views; **D**. Inflorescences; **E**. Part of inflorescence, showing peduncle, pedicel and bracts; **F**. Bracts; **G**. Calyx and disc; **H**. Flowers; **I**. Dissected corolla, showing stamens and pistil (Photos by Son Thanh Hoang).

**Table 1.** Morphological and ecological comparison of *Didymocarpus xuanlienensis*, *D. brevipedunculatus*, and *D. purpureobracteatus*.

Characters	*D. xuanlienensis*	*D. brevipedunculatus*	*D. purpureobracteatus*
**Petiole**	1.1–1.7 cm long	4.5–12.0 cm long	0.3–11.0 cm long
**Leaf blade shape**	Elliptic to oblong, base cuneate, apex acuminate	Ovate, base extremely obliquely cordate, apex attenuate to acuminate	Ovate to elliptic or obovate, base cuneate to cordate, apex acute to acuminate
**Leaf blade indumentum**	Adaxially and abaxially appressed pubescent	Adaxially densely villous with eglandular, multicellular hairs, abaxially densely villous with eglandular, multicellular hairs along veins	Adaxially sparsely appressed puberulent to glabrous, abaxially puberulent to nearly glabrous along veins
**Peduncle**	Glandular-pubescent	Densely villous with eglandular, multicellular hairs	Glabrous
**Bract**	Green, broadly ovate, glabrous	Green to light green, orbicular to ovate, sparsely villous with eglandular, multicellular hairs	Purple, often connate at base, ovate to elliptic-ovate, glabrous
**Calyx**	Red to light green outside, actinomorphic, broadly campanulate, ca. 5 mm long; lobes triangular	Often light green, sometimes purplish outside, campanulate, 6–7 mm long; lobes ovate to semiorbicular	Purple, slightly zygomorphic, tubular campanulate, 10–12 mm long; lobes semiorbicular
**Corolla**	3.3–3.9 cm long, white, with 15 longitudinal pink striations	4.0–4.5 cm long, white, inside with nine purplish to deep red longitudinal stripes	3.2–4.5 cm long, purple to pinkish purple with darker stripes
**Distribution and habitat**	Thanh Hoa, Vietnam, ca. 574 m a.s.l.	Yunnan, China, ca. 1200 m a.s.l.	Yunnan, China, 1400–2200 m a.s.l.
**Phenology**	Flowering October–December	Flowering August–September	Flowering August–September

#### Description.

***Herbs*** perennial, lithophytic, up to 50 cm tall. Stems erect, appressed pubescent. ***Leaves*** opposite; petiole 1.1–1.7 cm long, appressed pubescent; blade herbaceous, elliptic to oblong, 12.5–19.5 × 5.5–7 cm, base cuneate, often oblique, apex acuminate, margin serrate to subentire, adaxially dark green, appressed pubescent, abaxially light green, appressed pubescent; venation pinnate, lateral veins 7–9 pairs. ***Inflorescences*** terminal or subterminal, cymose, 7–15-flowered; peduncles 3.5–6 cm long, glandular-pubescent; bracts paired, green, broadly ovate, 3–5 mm long, 4–6.5 mm wide, glabrous on both surfaces. ***Pedicel*** 4–18 mm long, glandular-pubescent. ***Calyx*** actinomorphic, broadly campanulate, ca. 5 mm long, shallowly 5-lobed, outside red to light green, glabrous, inside light green; lobes bent outward, equal, triangular, ca. 1.5 mm long, 2.5 mm wide. ***Corolla*** 3.3–3.9 cm long, white, with 15 longitudinal pink striations extending from corolla tube to lobes, glabrous on both surfaces; tube infundibuliform-tubular, 2.1–2.6 cm long; upper lip 2-lobed, lobes round, ca. 4 mm long, 6 mm wide; lower lip 3-lobed, lobes subround, lateral lobes ca. 5 mm long, 6 mm wide, central lobe ca. 5 mm long, 6.5 mm wide. ***Stamens*** 2, adnate to ca. 1.5 cm above the corolla base; filaments ca. 1 cm long, glabrous; anthers coherent, ca. 3 mm long, pale yellow, villous. ***Staminodes*** 3, linear, lateral staminodes 2–4 mm long, central staminode ca. 3 mm long, adnate to 1.5–2 cm above the corolla base. ***Disc cupular***, ca. 2 mm high. ***Pistil*** 2.2–2.5 cm long; ovary linear, 1.8–2.1 cm long, glabrous; style 2–3 mm long, sparsely pubescent to subglabrous; stigma capitate. ***Capsules*** not seen.

#### Etymology.

The specific epithet “*xuanlienensis*” refers to the type locality in Xuan Lien National Park, Vietnam. A Vietnamese name is proposed here as “Song quả Xuân Liên”.

#### Phenology.

Flowering from October to December.

#### Distribution and habitat.

Currently, this species is known only from the type locality, where it grows on moist rocks within an evergreen forest at an elevation of ca. 574 m.

#### Conservation assessment.

Only a single population with fewer than 20 mature individuals of *Didymocarpus xuanlienensis* has been recorded to date. The extent of occurrence (EOO) is less than 100 km^2^, and the area of occupancy (AOO) is less than 10 km^2^. However, this population and its habitat are well protected within Xuan Lien National Park, with no signs of human disturbance, and are not expected to be subject to any human interference or destruction in the foreseeable future. Consequently, following the IUCN Red List Categories and Criteria (IUCN Standards and Petitions Committee 2024), its threat status is currently assessed as “Critically Endangered” (CR) (B1B2aD).

##### Key to the species of *Didymocarpus* in Vietnam

**Table:** 

1	Ovary glabrous	**2**
–	Ovary pubescent	**5**
2	Leaf blade near round or broadly elliptic	***D. kerrii***
–	Leaf blade ovate, elliptic, or oblong	**3**
3	Corolla pubescent outside	***D. punduanus* var. *pulcher***
–	Corolla glabrous on both surfaces	**4**
4	Leaf blade apex acute; bract rounded, connate at base; corolla 2.6–3.4 cm long, red	***D. tamdaoensis***
–	Leaf blade apex acuminate; bract broadly ovate, free; corolla 3.3–3.9 cm long, white with pink striations	***D. xuanlienensis***
5	Bract lanceolate or oblong-lanceolate	**6**
–	Bract ovate, elliptic ovate, or orbicular	**7**
6	Base of leaf often axisymmetric; bract 2–3 mm long	***D. poilanei***
–	Base of leaf asymmetric; bract 4–5 mm long	***D. phuquocensis***
7	Bract less than 3 mm long; corolla white, less than 3 cm long	***D. dalatensis***
–	Bract more than 3 mm long; corolla pinkish-purple to blackish-purple, more than 3 cm long	**8**
8	Peduncle glabrous; bracts connate at base; calyx 1–1.2 cm long; corolla purple to pinkish-purple with darker stripes	***D. purpureobracteatus***
–	Peduncle glandular hairy; bracts separate; calyx 6 mm long; corolla blackish-purple	***D. puhoatensis***

## Discussion

The karst area in southern China and northern Vietnam is well known for its high levels of biodiversity and endemism (Zhu 2007). The discovery of *Didymocarpus xuanlienensis* in northern Vietnam highlights the Indochina-China transition zone as a critical hotspot for Gesneriaceae diversity (Wang et al. 1998; Tan et al. 2020, 2025; Wei et al. 2022). This finding aligns with recent studies that reveal congruent spatial patterns of species richness and phylogenetic diversity in karst flora (Xu et al. 2021). The high levels of diversity and endemism within *Didymocarpus* likely stem from an explosive radiation of gesneriad lineages during the Miocene, a process that was further shaped by paleoclimatic fluctuations in southern China and Indochina, which acted as major drivers of speciation in these specialized karst habitats (Kong et al. 2022; Tan et al. 2026).

The new species grows on moist rocks within karst evergreen forests, exhibiting the specialized lithophytic habit common among many Gesneriaceae species (Wang et al. 1998; Tan et al. 2020). Unfortunately, karst landscapes worldwide are facing increasing threats from both climate change and human activities (Clements et al. 2006). Known from only a single population, this new taxon underscores the need for continued field surveys in under-explored national parks. In addition, integrating this new record into comprehensive regional checklists is essential for maintaining accurate knowledge of the geographical distribution of Gesneriaceae across the Asian tropics.

## Supplementary Material

XML Treatment for Didymocarpus xuanlienensis

## Additional information

### Artificial Intelligence (AI) use

The authors accept full responsibility for the content of the manuscript, including the disclosure of any use of AI.

No AI tools were used in the preparation of this manuscript.

### Author ORCIDs

V.T.T. Tran https://orcid.org/0009-0005-1065-9639

S.T. Hoang https://orcid.org/0000-0003-0351-929X

W.-G. Wang https://orcid.org/0000-0002-7372-147X

X.-A. Lang https://orcid.org/0000-0001-5362-9580

H.-C. Xi https://orcid.org/0009-0006-0909-6004

### Data availability

All of the data that support the findings of this study are available in the main text.
